# Regulator experiences of trials during Ebola epidemics in Sierra Leone, Guinea, and the Democratic Republic of the Congo

**DOI:** 10.1111/tmi.14111

**Published:** 2025-04-03

**Authors:** Kambale Kasonia, Frank Baiden, Frédéric Le Marcis, Bruno Lapika, Joel Kiyulu, Henri Kimina, Freddy Bikioli, Fanny Attas, Anthony Mansaray, Rose Burns, Elysée Nouvet, Séverine Thys, Antea Paviotti, Daniela Manno, Mukeh Kenneth Fahnbulleh, Bailah Leigh, Mohamed Samai, Brian Greenwood, Shelley Lees, Patrick Mitashi Mulopo, Deborah Watson‐Jones

**Affiliations:** ^1^ London School of Hygiene & Tropical Medicine London UK; ^2^ University of Health and Allied Sciences Ho Ghana; ^3^ Ecole Normale supérieure de Lyon Lyon France; ^4^ Université de Montpellier, Institut de Recherche pour le Développement, IRD Institut National de la Sante et de la Recherche Médicale, INSERM Montpellier France; ^5^ Département de Sciences Sociales, Centre de recherche et de formation en infectiologie de Guinée CERFIG Republic of Guinea; ^6^ Département d'Anthropologie Université de Kinshasa Kinshasa The Democratic Republic of the Congo; ^7^ Tropical Medicine Department Université de Kinshasa Kinshasa The Democratic Republic of the Congo; ^8^ School of Health Studies Western University London Canada; ^9^ Centre de Coopération Internationale en Recherche Agronomique pour le Développement, CIRAD Montpellier France; ^10^ University of Antwerp Antwerp Belgium; ^11^ Ministry of Health Freetown Sierra Leone; ^12^ College of Medicine and Allied Health Sciences University of Sierra Leone Freetown Sierra Leone; ^13^ Mwanza Intervention Trials Unit National Institute for Medical Research Mwanza Tanzania

**Keywords:** Committee, Ebola, Epidemic, Ethics, Regulators

## Abstract

**Introduction:**

During the 2014–2016 Ebola epidemic in West Africa and the Ebola outbreaks between 2018 and 2020 in the Democratic Republic of Congo, vaccines and other tools for prevention and treatment had to be taken through trials in exceptional circumstances using accelerated processes.

**Materials and methods:**

We interviewed members of ethics committees, health authorities, health professionals, and political authorities in the Democratic Republic of Congo in 2021 and held a workshop with ethics committee members and regulatory authorities from Sierra Leone and Guinea in 2022 in order to document their experiences of reviewing, approving, and regulating current and new studies during epidemics and outbreaks, and to document lessons learnt and their recommendations for the rapid review of clinical trial protocols during public health emergencies.

**Results:**

Similar barriers were identified in the three countries. These were related to weak legal frameworks and partnerships between ethics committees and regulatory bodies. Inadequate human resources, outdated standard operating procedures and guidelines, and lack of finance to support timely reviews were identified. We also noted a lack of awareness from politicians, scientists, and communities about the existence and functions of regulatory bodies/ethics committees, a lack of independence, and low interest in research. Opportunities identified by the institutions in the countries concerned included training ethics committee members and networking with experienced international platforms like the African Vaccine Regulatory Forum. Laws on regulating research have been updated in Sierra Leone and in Guinea, but not yet in the Democratic Republic of Congo.

**Conclusion:**

Regulatory bodies have been facing many challenges in terms of a lack of a legal framework, a lack of finance, and a lack of support from politicians, scientists, and communities. Networking has been an opportunity for these regulators to mitigate these impediments.

## INTRODUCTION

There have been at least 15 documented outbreaks of Ebola virus disease (EVD) in sub‐Saharan Africa over the past 10 years [1]. The Ebola outbreak in West Africa in 2014–2016 was the most devasting to date, resulting in 11,310 deaths in Guinea, Liberia and Sierra Leone [2]. It drew unprecedented attention on the need to find effective tools for EVD prevention and treatment, requiring EVD vaccine trials during this and subsequent EVD outbreaks. A call was then made for the use of experimental interventions to try to contain the outbreak (compassionate use) [3]. Ethical dilemmas about randomization to control arms for a deadly disease were raised among many other concerns [3, 4, 5, 6]. Local and international vaccine trial regulators responsible for evaluating these trial protocols did so under huge time pressure with limited resources and without clear precedent [7, 8]. Ebola trial participants experience during the Ebola outbreak in West Africa including ethical dilemmas surrounding “participants payments being at the very bottom of the clinical research pay scale”, “participants feeling left behind when the financial benefits of vaccine development will be shared” as seen in the perspective of trial participants have been published [9]. The impact of trial implementation on the quality of care within Ebola Treatment Centres has been discussed [10]. WHO ethics review committee experiences during the West Africa Ebola epidemic have already been published [11]. These experiences led to recommendations on how to accelerate study approvals in future public health emergencies [11]. In this study, we set out to learn regulators experiences during these Ebola outbreaks. The EVD trials were the first phase 4 vaccine trials to be initiated within an ongoing public health emergency caused by that pathogen. Prophylactic EVD vaccines were subsequently licensed by the European Medicines Agency (Zabdeno® and Mvabea®) and the United States Food and Drug Administration (Ervebo®) and given World Health Organisation (WHO) prequalification [12, 13]. The rapid containment of the more recent Ebola outbreak in Guinea in 2021 was due in part to the rapid deployment of currently available vaccines [14]. Recognising and appreciating how trial regulators (including ethics committees) in West Africa worked during the EVD outbreak of 2014–2016 provides important lessons for preparedness and effective vaccine trial regulation in future outbreaks of Ebola or other epidemic pathogens. The theoretical framework developed by the WHO ethics review committee on how to accelerate study approvals in future public health emergencies [11] guided the design and the analysis of our qualitative research.

Information about regulatory development presented in this article derives from two sources: [1] results from a workshop conducted in 2022 with members of the regulatory authorities of Sierra Leone and Guinea who were responsible for reviewing new studies during the 2014–2016 Ebola outbreak, and [2] interviews conducted in 2021 with members of ethics committees, health authorities, health professionals, and political authorities in the Democratic Republic of Congo (DRC). The objectives of both the workshop and the interviews were to summarise regulatory experiences of vaccine trials, to identify barriers and opportunities relating to the regulation of vaccine trials during outbreaks, and to make recommendations to assist both researchers and regulators in ensuring timely and high‐quality reviews during future outbreaks.

## METHODS

### Guinea and Sierra Leone

A qualitative approach to addressing some of the challenges considered above included a two‐day workshop organised by the London School of Hygiene and Tropical Medicine (LSHTM), the *Institut National de la Santé et de la Recherche Médicale* (INSERM), and the *Centre de Recherche et de Formation en Infectiologie de Guinée* (CERFIG) held in March 2022 in Conakry at the Gamal Abdel Nasser University. The workshop was attended by 39 representatives of institutions in Guinea and Sierra Leone who were responsible for research, ethics reviews of research protocols, medicine and vaccines regulation, and health security during the EVD outbreak in 2014–2016. All the participants invited to the workshop attended the workshop and actively participated. Participants included regulators and researchers involved in research or study review during the EVD outbreak in 2014–2016 (Table S1). Discussions were held in French (for Guinea participants) and in English (for Sierra Leone participants). Discussions were recorded. Two research fellows (one fluent in French and one fluent in English) took notes about the discussions and summarised key points from discussions. Then the summary of the recordings was crosschecked by workshop participants after the sessions. Discussions were stopped by the moderator when they reached saturation. The summary was validated after reaching consensus from the workshop participants about its content. No ethics approval for the workshop agenda and topic guides was sought. Verbal consent was requested from participants to permit publication of their views and recorded feedback.

The workshop combined brief keynote addresses, structured group, and plenary discussions with simultaneous English and French translations. Questions to guide these discussions (Table S2) were prepared by the international organising committee, all of whom had expertise in experience and research in the context of public health emergencies in West Africa (EN, FBa DM, AM, DWJ, RB, BG, BL, MS, FLM, KK). Participants could attend face‐to‐face or online via Zoom. Deductive analysis was then done using the theoretical framework built from the WHO ethics review committee recommendations [11]. The theoretical framework approach was the driving force in our analysis. In this theoretical framework, we considered the forces reducing the functionality of regulatory bodies and the forces supporting a good performance of regulatory bodies in the context of the deadly Ebola outbreak experienced in West Africa and in the DRC. Most of these influences were grouped under topics such as researchers, communities, or collaborations among regulators.

### DRC

Using a qualitative study approach, 32 face‐to‐face individual interviews were conducted with members of Congolese ethics committees (ECs) and regulatory bodies between May and June 2021 in Kinshasa, Mbandaka, and Boende, DRC.

Interviews were recorded and transcribed. Interview guides focused on experiences of DRC regulatory and ethics authorities during Ebola outbreaks (Table S3). Participants included members of ECs, health authorities, health professionals, and political authorities involved in the 2017 (8th) and 2018 (9th) Ebola outbreaks in Bas Uele and Equateur respectively (Table S4). All participants approached agreed to participate. We stopped the interview when saturation was reached. Study design and data collection were done by PMM, ST, BL, JK, and HK, and deductive data analysis by FBi and AP, which was undertaken separately from the analysis in the other sites. The theoretical framework approach we used remained the driving force Ethical approval of the research protocol and interview guides was granted by the DRC National Committee of Health Ethics (CNES – *Comité National d'Éthique de la Santé*) (Avis No. 253/CNES/BN/PMMF/2021 du 11/05/2021).

## RESULTS

### Guinea and Sierra Leone workshop

Insights shared by research ethics' committee members and regulatory authorities in attendance at the workshop are presented below according to the principal topics discussed: regulatory and ethics reviews pre‐ and during the Ebola outbreak, barriers and opportunities relating to the regulation of vaccine trials during Ebola outbreaks, and recommendations.

### Regulatory and ethics reviews pre‐ and during the Ebola outbreak

Prior to the Ebola outbreak, research ethics review and regulation activities for medicine or vaccine trials were challenging in Sierra Leone and in Guinea. The Sierra Leone Ethics and Scientific Review Committee (SLESRC) generally reviewed 8–12 non‐clinical trial research protocols per month before the Ebola outbreak, with this number rising to between 20 and 25 protocols per month during the outbreak. Reviews for clinical trial protocols for medicines and vaccines by the Pharmacy Board of Sierra Leone (PBSL), the national regulatory authority, also rose in this period, by one to two protocols per month.

In Guinea, before the Ebola outbreak, the *Comité National de la Recherche en Santé, CNES* (Guinea National Ethics Committee) only reviewed 9–10 protocols per annum. With the advent of the Ebola outbreak, that number grew rapidly to 24 protocols in 2014 and 76 in 2015. In terms of regulatory oversight, there was no functional technical committee at the level of the DNPM (*Direction Nationale de la Pharmacie et du Médicament, Ministère de la Santé*) when the first Ebola outbreak started in Guinea.

As described below, during the Ebola outbreak, several training courses were organised to strengthen the capacities of the ethics and regulatory institutions to review clinical trial protocols in Guinea and Sierra Leone. Both ethics and regulatory authorities took advantage of the platform created by the African Vaccine Regulatory Forum (AVAREF) to receive training and to conduct joint reviews of protocols in Guinea and Sierra Leone [15]. The other platforms that were identified as contributing to capacity strengthening included the West African Consortium for Clinical Research on Epidemic Pathogens (WAC‐CREP) [16] and the RAFES, *Réseau Africain Francophone d'Ethique en Santé* (African Francophone Health Ethics Network) [17] for the Guinea National Ethics Committee, CNES. The participants found these training programmes beneficial.

### Barriers and opportunities relating to the regulation of vaccine trials during Ebola outbreaks

#### Sierra Leone

In Sierra Leone, key barriers to ethics oversight and regulation by the medicine regulatory authority included limited policies and laws to govern clinical trials, limited resources for ECs, and poor communication between ethic committees and medicine regulatory authorities. As one workshop participant noted:

“The requirements for approval and regulation by the various agencies were not backed by robust laws. This undermined consistency in the application of guidelines. Without laws, investigators and sponsors could not be sanctioned. It also made the procedures vulnerable to undue influences from political and ministerial authorities.” (Sierra Leone participant).

In Sierra Leone, ethics committees (ECs) and regulators performed their functions without consulting each other because there had been little or no need for the two agencies to collaborate previously. They rarely engaged with institutions beyond the health ministries. The urgency of the situation required close collaboration between the ethics and regulatory agencies, but there was no blueprint to guide such engagements. Decisions were therefore ad hoc and guided by the needs of the emergency. Members of EC and regulatory bodies had not received formal training on their role in vaccine trial regulation. With little to no government funding, regulatory agencies in Sierra Leone and the Sierra Leone Ethics and Scientific Review Committee (SLESRC) depended almost entirely on fees paid by investigators and sponsors. The EC in Sierra Leone lacked permanent offices, and meetings held at ad hoc locations affected record keeping (with no precise location for long‐term record keeping).

The workshop participants observed that opportunities in Sierra Leone for a more effective response have been supported by the recent establishment of a legal and policy framework for the regulation of vaccine trials in the new Public Health Act 2023. Guidelines and standard operating procedures were revised by the government in order to include: recognition of a public health emergency and its implications for the regulation of clinical trials; considerations for expedited review; sharing information with local and international bodies; simultaneous submission of protocols to ethics and drug regulatory agencies; collaborative review of protocols; acceleration of procedures for importing investigational products and exporting biological samples; promotion of local capacity building through the emphasis on local co‐leadership of clinical trials and the inclusion of at least one local clinical trial associate in the trial monitoring team; recognition of the role of information communication and technology; and provision for the online submission of research protocols. Networking has provided a big opportunity for collaboration and training: the PBSL regulatory authority in Sierra Leone became part of the partners of the German Federal Ministry of Health (*Bundesministerium für Gesundheit, BMG*) Global Health Protection Program (GHPP) in terms of regulatory training [18], and both PBSL and the Sierra Leone Ethics and Scientific Review Committee (SLESRC) have partnered with the King's Centre for Global Health and Health Partnerships in training and support [19].

#### Guinea

Guinean participants described several of their country's barriers to effective regulation of clinical studies during the EVD outbreak. Echoing their Sierra Leone colleagues, a Guinea participant emphasised a lack of policies and processes as the outbreak took grip of the country:

“The Guinea National Ethics Committee and the regulatory authority were unprepared to deal with the 2014‐2016 EVD outbreak. The parliament was not familiar with the work of ethics committees and how the regulatory bodies operate. There were no procedures for vaccine licensing. Guinea regulatory bodies were not supervising clinical trial sites for various reasons, particularly a lack of procedures. There were no laws regulating material transfer agreements. For this reason, all the samples collected in research studies during the outbreak went outside Guinea, leaving local researchers without any samples for their research.” (Guinea participant)

Also aligned with the descriptions shared from Sierra Leone, Guinean participants highlighted the lack of synergy between advisory and legislative bodies, with key decision‐making bodies at times identifying priorities in isolation. This contributed to delays in setting national health priorities during the Ebola outbreak in the country.

Participants from Guinea pointed out the challenge of ensuring informed consent in emergency situations, as well as managing ethical issues related to scientific methodological requirements such as randomisation that included a placebo control arm for a deadly disease.

“By that time, only supportive treatment was available, and the potential study participant had very little choice in terms of refusing to participate in this trial on therapeutic candidates (“empty choice”).” (Guinea participant)

“The investigators were claiming that they needed a control arm to get valid efficacy and safety results. On the other hand, this raised the ethical issue of randomising participants to a placebo treatment for a disease with a high mortality rate. When the compassionate use of vaccines against Ebola was put in place, already approved protocols continued to use their placebo control arms while Ebola vaccines were given to other people exposed to the same risk as compassionate treatment.” (Guinea participant)

Echoing the situation in Sierra Leone, participants noted the lack of state financial support for the EC. The participants also reported that the staff trained through local capacity building during clinical trials were not integrated into the local academic institutions at the end of the clinical trials.

“Trial sponsors have been collaborating with professors in Guinea without involving Guinean universities as research bodies. This resulted in the lack of sustained research in Guinea, which meant that after a clinical trial is completed, the research site is sometimes dismantled.” (Guinea participant).

Guinean participants identified the following important improvements to the public health emergency regulatory environment that had emerged during or after the 2014–2016 outbreak. They noted the update of the pharmaceutical law L‐2018/024/AN on medicines, medical products, and the practice of the profession of pharmacist in Guinea in 2018 specified the medicines regulatory authority's role. The adopted practice of visiting research sites during the Ebola outbreak after researchers understood the role of ECs and medicines regulatory authorities was mentioned. The sub‐regional cooperation (West Africa Consortium for Clinical Research on Epidemic Pathogens, WAC‐CREP) for training of EC and medicines regulatory authorities and collaboration was put in place in July 2015. The implementation of online meetings for expediting protocol reviews during Ebola outbreaks was reported. The skills transfer/students training and research in clinical trials was counted among the post‐outbreak benefits to the country. The greater recognition by researchers of the importance of EC reviews after training of researchers and regulatory authorities was also reported as an important achievement. The provision of free healthcare to study participants during clinical studies was framed as being important to participants' safety and wellbeing as access to healthcare is not always guaranteed in developing countries. Guinean participants from the national EC explained that the EC had decided to make it mandatory for submitted research proposals to include community engagement staff members following previous incidents between communities and research teams at research sites.

### DRC

#### Regulatory and ethics review experiences of vaccine trials during Ebola outbreaks

Before the 2017 (8th) and 2018 (9th) Ebola outbreaks in the country in Bas Uele and Equateur respectively, study protocols were not frequently submitted to the ECs. Representatives from the DRC national ethics committee (CNES) and the University of Kinshasa School of Public Health ethics committee (ESP CE) reported an increase in protocol submissions following these outbreaks, although no numbers were provided. Protocols submitted to the Protestant University of the Congo ethics committee (UPC EC) have focused mainly on studies in family medicine, child and maternal health, chronic diseases, malaria, and post‐vaccination reactions. At the time of the interviews, the UPC EC had never evaluated Ebola‐related protocols, although it had evaluated the protocols for two vaccine trials on hepatitis B and human African trypanosomiasis.

During and after the 2017 and 2018 Ebola outbreaks, the increased demand and urgency for ethical clearance of study protocols led ECs to drastically accelerate their review processes. During the 8th outbreak in Bas Uele, the ESP EC was able to grant ethical clearance in one or two days, while reviews under non‐emergency circumstances previously required 30 days. At the CNES, review timelines likewise were reduced from 30 to 14 days during the two outbreaks. It was noted by the president of the CNES that the AVAREF (African Vaccine Regulatory Forum) today requires ECs to review protocols within 14 days in emergencies.

#### Barriers relating to the regulation and ethics review of vaccine trials during Ebola outbreaks

Key barriers highlighted by DRC study participants included: difficulties monitoring trial activities in the field; outdated guidelines; lack of trained EC staff; and the “poor quality” of protocols submitted during the Ebola and Covid‐19 outbreaks. Protocols that did not respect the most basic ethics requirements had to be revised, delaying approval for studies that needed to be rapidly implemented during an outbreak.

One participant mentioned that the government allowed samples to be exported without monitoring the samples storage conditions, manipulation, and transport conditions, and that it did not make any efforts to ensure proper infrastructure in the country for sample storage and analysis in situ instead of sending them abroad. The guidelines for the ethical evaluation of research involving human subjects in the DRC date back to 2011 [20]. Several participants said that these were not detailed enough as they lacked specific instructions for research under emergency situations, despite several Ebola outbreaks since 2011.

Interviewees also described how researchers who were submitting protocols perceived the activities of the DRC ECs. Firstly, ECs were sometimes perceived as mere administrative bodies and ethical clearance as an administrative formality. According to one of the interviewees, there was a poor understanding of the importance of ethics and ethical review, especially among non‐medical experts. The CNES complained that sometimes applicants had started their study before submitting their protocol for review. Secondly, ECs were perceived as policing bodies, aiming to “punish” or to “block” the research. According to the CNES, the reason for this perception was the research applicants' lack of training in ethics. Thirdly, ECs were sometimes perceived as being influenced by politicians and sponsors. According to one of the interviewees, ECs in the country are incapable of countering decisions taken by political representatives and sometimes inclined to approve protocols to investigators or sponsors that provided resources for field visits or other types of financial benefits, since ECs' members work on a voluntary basis and are not paid by the state. Experts' technical advice was sometimes perceived as political because of people's general lack of trust in the political authorities and the political system in the DRC.

#### Best practices and opportunities relating to the regulation of vaccine trials during Ebola outbreaks

Participants observed that during Ebola outbreaks, ECs gained experience in reviewing protocols.

Several types of cross‐country collaborations took place during and after the Ebola outbreaks, allowing regulatory bodies to share their experiences and improve their skills. Today, the CNES is represented at the African Vaccine Regulatory Forum (AVAREF) and benefits from the support of the European and Developing Countries Clinical Trials Partnership (EDCTP). The CNES is also involved in the EC for Health Research in Central Africa (*Comité d'éthique pour la Recherche en Santé en Afrique Centrale*; CERSAC) and in the African Francophone Network of Health Ethics (*Réseau Africain Francophone d'éthique en santé*; RAFES). The *Autorité Congolaise de la Réglementation Pharmaceutique* (ACOREP), which is the DRC's medicines regulatory body, is represented at the sub‐regional platform of the SADC (Southern African Development Community) member states for joint evaluations of medicines, vaccines, and biological products.

### Recommendations

Guinea and Sierra Leone workshop participants agreed on the following recommendations for ethics review of future public health emergency research studies: the necessity of establishing procedures (e.g. Standard operating procedures, policies and memoranda of understanding) for networking among regulatory bodies and ECs at a national and international level; systematisation of the monitoring of clinical trial sites during the study; strengthening of communication between ECs and regulatory authorities in the management of adverse events; training of EC members, especially new members; building a collaborative and trusting relationship between the Guinea National Ethics Committee and the SLESRC with the Health Committee of their respective parliaments before any new emergency; involvement of the national research institutions in the research programmes developed by international study sponsors; and a requirement for dissemination of clinical trial results to trial participants.

The following recommendations are based on the DRC interview data: investing in the creation of a functional cooperation between the bodies involved in clinical trials (ECs, directorate of laboratories, and ACOREP);investing in training for members of the ECs to reinforce their capability to work in emergency situations; investing in the ability of ECs to allow them to conduct field monitoring so as to avoid any possible bias when their field visits are funded by the applicants; developing opportunities for sharing experiences among different ECs and other national and international stakeholders to allow them to learn from each other and thus improve their skills; networking to make ECs more visible; updating the guidelines for the ethical evaluation of research involving human subjects in the DRC to include emergency scenarios and making them in line with the AVAREF guidelines for joint and assisted reviews of clinical trials applications [21].

## DISCUSSION

The aim of our study was to explore the experiences of regulators during the 2014–2016 Ebola epidemic in West Africa and the subsequent Ebola outbreaks between 2018 and 2020 in DRC. We set out to identify barriers and opportunities relating to the regulation of vaccine trials during outbreaks and to make recommendations to assist both researchers and regulators in ensuring timely and high‐quality reviews during future outbreaks.

The weaknesses identified by participants included undue influence from political and ministerial authorities. Ethics committees are meant to be independent as set out in the internationally accepted guidelines like the WHO's standards and operational guidance for ethics review of health‐related research with human participant [22], the CIOMS international ethical guidelines for health‐related research involving humans [23], and the integrated addendum to ICH E6(R1) guideline for good clinical practice [24] among others. In the WHO's standards and operational guidance for ethics review of health‐related research with human participant, it is stressed that authorities should ensure that ethics committee reviews are done in an independent manner. An ethic committee under political influence puts into question its independence. It was reported that operation of the regulatory authorities in Sierra Leone and the DRC depended almost entirely on fees paid by the investigators and the sponsors. The DRC interviewees pointed out that ECs in that country are incapable of countering decisions taken by political representatives and sometimes are inclined to approve protocols for investigators or sponsors that provided resources for field visits or other types of financial benefits, since ECs' members work on a voluntary basis and are not paid by the state. The CIOMS international ethical guidelines for health‐related research involving humans (prepared in collaboration with WHO) clearly state that: “independent scientific and ethical review is critical to engender community trust for research” [23].

The integrated addendum to ICH E6(R1): guideline for good clinical practice (3.2.2) recommends that each ethics committee should maintain written records of its activities and meetings [24]. Participants reported that the ethics committee in Sierra Leone lacked offices where they could organise record keeping. Thus, researchers interested in understanding how reviews were done in Sierra Leone during the 2014–2016 Ebola epidemic in West Africa will face the absence of complete records able to provide valuable information on the decisions and the processes conducted. Despite the challenges faced by the regulators, like the lack of financial support, the regulators (working on a voluntary basis without salary) were able to organise online meetings to expedite the review of protocols during the Ebola outbreak. The time for review in the DRC was halved from 30 days to 14 days. Under similar pressure during the 2014–2016 West Africa Ebola outbreak, the MSF (Médecins Sans Frontières) ethics review board was able to make reviews in an average of 12.4 days [8].

The DRC and the Guinea regulatory authorities noted the poor quality of some protocols submitted by investigators. Poor quality prolonged the review of these protocols. Lessons learnt from the MSF ethics review board during the West Africa Ebola outbreak have led to the recommendation that generic protocols should be prepared in advance to accelerate their approval when facing future health emergencies [8].

Absence of laws governing material transfer agreements led to samples from clinical trials being sent outside Guinea, leaving local researchers without any samples for study, as reported by a Guinea participant. An estimated 80,000 specimens were sent outside the area affected by the West Africa Ebola outbreak, and their precise location is unknown [25] despite the CIOMS international ethical guidelines for health‐related research involving humans (prepared in collaboration with WHO) stating that: “if the specimen and data are stored outside the original setting, there should be provision to return all material to that setting and share possible results and benefits” [23].

This study had a number of weaknesses.

We invited only one ethics committee member from Sierra Leone and our study did not encompass Liberia because Liberia was not among the countries covered by the EBOVAC 3 project framework which supported this study [26]. We also acknowledge that recall bias must have affected our results because the workshop was held 6 years following the end of the West Africa EVD outbreak.

The summaries made were analysed using a specific theoretical framework. This approach left out other information assessed as not contributing to the predefined theoretical framework.

Ethics reviews and their related ethical dilemmas are numerous and single workshop and series of interviews cannot embrace and cover them all. Topics the participants did not raise like “ensuring free and informed consent in a public health crisis”, “the risk of therapeutic misconception”, and the “allocation of scarce research resources” could be the object of future research.

## CONCLUSIONS

The study presented provided an opportunity to understand the experiences and challenges faced by ECs and regulators in three of the countries most affected by the largest Ebola outbreak globally, which increased the demand for urgent protocol reviews of often complex studies and trials. All three countries reported significant barriers to effective reviews and regulation of clinical studies and trials. However, the outbreaks provided an opportunity to address some of these barriers, including training and networking with platforms like AVAREF, and modification of laws governing clinical trials regulation. Challenges remain in terms of updating the existing guidelines in DRC and funding ECs and medicines regulatory authorities.

## FUNDING INFORMATION

This project has received funding from the Innovative Medicines Initiative (https://www.imi.europa.eu) 2 Joint Undertaking under grant agreement no. 800176. This Joint Undertaking receives support from the European Union's Horizon 2020 research and innovation programme (https://commission.europa.eu/funding‐tenders/find‐funding/eu‐funding‐programmes/horizon‐europe_en) and the European Federation of Pharmaceutical Industries and Associations (https://www.efpia.eu). DWJ was awarded this grant for the EBOVAC3 consortium. Funders did not play any role in the study design, data collection and analysis, decision to publish, or preparation of the manuscript.

## CONFLICT OF INTEREST STATEMENT

The authors have declared no competing interests.

## Supporting information


**TABLE S1:** Workshop participants.

**TABLE S2:** Topic guides used during the workshop.

**TABLE S3:** Interview guides DRC.

**TABLE S4:** List of individual interviews DRC.

## Data Availability

Data cannot be shared publicly because of confidentiality. The participants did not grant us permission to share publicly their identity and the recordings of the interviews and the workshop. Workshop topic guides and interview guides have been added as supporting information files.
