# Supplementary material for: Regulator experiences of trials during Ebola epidemics in Sierra Leone, Guinea, and the Democratic Republic of the Congo
Source: Trop Med Int Health. 2025 Apr 3;30(6):539–46. doi: 10.1111/tmi.14111 (PMC12136929; doi:10.1111/tmi.14111)
Supplement: Supplementary file 1 — TABLE S1: Workshop participants. [file TMI-30-539-s004.docx]

# **Supplementary Table 1 – List of workshop participants**

**Participants from Guinea**

| **No** | **Country** | **Category** | **Name of Institution** | **Role** |
| --- | --- | --- | --- | --- |
| 1 | Guinea | Researcher | The Alliance for Medical Action (ALIMA) | Head of the ALIMA mission in Guinea  (*Chef de Mission ALIMA en Guinée*) |
| 2 | Guinea | Researcher | ALIMA | Alima Project coordinator in EBOVAC3 (*Coordinateur projet Alima dans EBOVAC3*) |
| 3 | Guinea | Researcher | Institut National de la Sante et de la Recherche Médicale (INSERM)/ALIMA | Head of INSERM/ALIMA socio-anthropological research in EBOVAC3 (*Responsable de la recherche socio-anthropologique INSERM/ALIMA dans EBOVAC3*) |
| 4 | Guinea | Researcher | INSERM | Inserm Project Leader (on the Guinea side) in EBOVAC 3  [*Chef de Projet Inserm (du côté Guinée) dans EBOVAC 3*] |
| 5 | Guinea | Researcher | INSERM | Field coordinator of the EBOVAC 3 clinical project and INSERM representative in Guinea  (*Coordinateur terrain projet clinique dans EBOVAC 3 et représentant de l'INSERM en Guinée*) |
| 6 | Guinea | Researcher | INSERM | Pharmacy Coordinator in EBOVAC 3  (*Coordinateur Pharmacie dans EBOVAC 3*) |
| 7 | Guinea | Researcher | INSERM | EUCLID Executive Director  (*Directrice Exécutive EUCLID*) |
| 8 | Guinea | Researcher | INSERM | INSERM project leader in EBOVAC 3 (Principal referent for the study)  [*Chef de projet INSERM dans EBOVAC 3 (Principale référente de l'étude)]* |
| 9 | Guinea | Researcher | INSERM | International Project Coordinator INSERM |
| 10 | Guinea | Researcher | Centre de Recherche et de Formation en Infectiologie de Guinée (CERFIG) | Head of clinical trials at the Centre de Recherche et de Formation en Infectiologie de Guinée (CERFIG) and Head of the Department of Infectious Diseases at the Donka National Hospital.  (*Chef des études cliniques au Centre de Recherche et de Formation en Infectiologie de Guinée (CERFIG) et Chef du Service des Maladies Infectieuses de l'Hôpital National Donka*) |
| 11 | Guinea | Researcher | African center for Excellence  [*Centre d’excellence Africain (CEA)]* | Researcher and Teacher at the African center for Excellence  (*Enseignant-chercheur du centre d’excellence Africain*) |
| 12 | Guinea | Regulator | National agency for health security  *[Agence Nationale de Sécurité Sanitaire* (ANSS)] | Member of the National agency for health security  (*Membre de l’Agence Nationale de Sécurité Sanitaire_ANSS*) |
| 13 | Guinea | Regulator | ANSS | Member of the National agency for health security  (*Membre de l’Agence Nationale de Sécurité Sanitaire_ANSS*) |
| 14 | Guinea | Regulator | Ethics committee | Member of the Guinea National Ethics Committee  (*Comité National d'Éthique pour la Recherche en Santé de Guinée*) |
| 15 | Guinea | Regulator | Ethics committee | Member of the Guinea National Ethics Committee  (*Comité National d'Éthique pour la Recherche en Santé de Guinée*) |
| 16 | Guinea | Regulator | Ethics committee | Member of the Guinea National Ethics Committee  (*Comité National d'Éthique pour la Recherche en Santé de Guinée*) |
| 17 | Guinea | Regulator | Ethics committee | Member of the Guinea National Ethics Committee  (*Comité National d'Éthique pour la Recherche en Santé de Guinée*) |
| 18 | Guinea | Regulator | Ethics committee | Member of the Guinea National Ethics Committee  (*Comité National d'Éthique pour la Recherche en Santé de Guinée*) |
| 19 | Guinea | Regulator | Ethics committee | Member of the Guinea National Ethics Committee  (*Comité National d'Éthique pour la Recherche en Santé de Guinée*) |
| 20 | Guinea | Regulator | Ethics committee | Member of the Guinea National Ethics Committee  (*Comité National d'Éthique pour la Recherche en Santé de Guinée*) |
| 21 | Guinea | Regulator | Ethics committee | Member of the Guinea National Ethics Committee  (*Comité National d'Éthique pour la Recherche en Santé de Guinée*) |
| 22 | Guinea | Regulator | Ethics committee | Trainee at the Guinea National Ethics Committee  (*Comité National d'Éthique pour la Recherche en Santé de Guinée*) |
| 23 | Guinea | Scientific Advisory team | Scientific council | First Vice-President of the Scientific Council of Riposte Contre Covid-19 in Guinea  (*Premier Vice-Président du Conseil Scientifique de Riposte Contre Covid-19 en Guinée*) |
| 24 | Guinea | Regulator | Ministry of Health/ National Directorate of Pharmacy and Medicines  (*Ministère de la Santé/ Direction Nationale de la Pharmacie et du Médicament*) | Member of the National Directorate for Pharmacy and Medicines  (*Membre de la Direction Nationale de la Pharmacie et du Médicament*) |
| 25 | Guinea | Researcher | Health and Sustainable Development Foundation  [*Fondation Santé & Développement Durable (FOSAD)]* | Chairman of the Health & Sustainable Development Foundation, Executive Director of the Research Centre (CEFORPAG)  [*Président Fondation Sante & Développement Durable, Directeur Exécutif Centre de Recherche (CEFORPAG)]* |
| 26 | Guinea | Workshop organizing committee | CERFIG (Workshop organizing committee) | Anthropologist and Director of Research (IRD, ENS Lyon, CERFIG)  [*Anthropologue et Directeur de recherche (IRD, ENS de Lyon, CERFIG)]* |
| 27 | Guinea | Workshop organizing committee | CERFIG (Workshop organizing committee) | In charge of research support in SHS (IRB, CERFIG)  [*Chargée de soutien à la recherche en SHS (IRD, CERFIG)]* |

**Participants from Sierra Leone**

| **No** | **Country** | **Category** | **Institution** | **Function** |
| --- | --- | --- | --- | --- |
| 1 | Sierra Leone | Researcher | College of Medicine and Allied Health Sciences &  Ministry of Health and Sanitation | Provost and Deputy Vice-Chancellor  Deputy Chief Medical Officer-Clinical |
| 2 | Sierra Leone | Researcher | College of Medicine and Allied Health Sciences | Principal Investigator – EBOVAC3 |
| 3 | Sierra Leone | Researcher | College of Medicine and Allied Health Sciences | Research Administrative Officer |
| 4 | Sierra Leone | Researcher | College of Medicine and Allied Health Sciences | Research Finance Officer |
| 5 | Sierra Leone | Regulator | Ministry of Health and Sanitation | Program Manager, Public Health Emergencies Management Program |
| 6 | Sierra Leone | Regulator | Ministry of Health and Sanitation | Program Manager |
| 7 | Sierra Leone | Regulator | Pharmacy Board of Sierra Leone | Head of Clinical Pharmacovigilance |
| 8 | Sierra Leone | Regulator | Pharmacy Board of Sierra Leone | Senior Inspector |
| 9 | Sierra Leone | Regulator | Sierra Leone Ethics and Scientific Review Committee | Member |
| 10 | Democratic Republic of the Congo | Workshop organizing committee | London School of Hygiene and Tropical Medicine | Research Fellow |
| 11 | Sierra Leone | Workshop organizing committee | London School of Hygiene and Tropical Medicine | Research Fellow |
| 12 | Sierra Leone | Workshop organizing committee | London School of Hygiene and Tropical Medicine | Associate Professor and Trial Manager |
