# Supplementary material for: Regulator experiences of trials during Ebola epidemics in Sierra Leone, Guinea, and the Democratic Republic of the Congo
Source: Trop Med Int Health. 2025 Apr 3;30(6):539–46. doi: 10.1111/tmi.14111 (PMC12136929; doi:10.1111/tmi.14111)
Supplement: Supplementary file 2 — TABLE S2: Topic guides used during the workshop. [file TMI-30-539-s001.docx]

Supplementary table 2: **WORKSHOP TOPIC GUIDES | Experience of regulatory bodies during the Ebola outbreak and lessons for future outbreaks | 7^th^ – 8^th^ March 2022 | Conakry, Guinea**

| **Day 1: 7^th^ March 2022** | |
| --- | --- |
| 9:30-10:10 | **When EBOLA struck – Regulatory and ethics environment when the 2014-2016 Ebola epidemic started in West Africa** |
|  | 1. What was the context for the trials.  2. How many proposals did they have to review per annum before the Ebola outbreak.  3. How big were the committees?  4. How long did reviews take?  5. What was the experience of vaccine trials research reviews?  6. Did you feel adequately resourced and or supported to carry out what was expected of you? |
| 10:30-12:30 | **7 Key challenges encountered, and 7 key opportunities presented in vaccine trial regulation during the Ebola outbreak** |
|  | 1. Group discussion with note taking and summary for plenary presentation  2. Groups will elect their own rapporteurs. |
| 15:00-15:30 | **Reflections on working with WHO, AVAREF, local and international NGOs and negotiating local politics during the Ebola outbreak** |
|  | **Key questions to be addressed by speakers (NO NEED FOR POWERPOINT PRESENTATION)** |
|  | 1. How will you describe the role played by local stakeholders to facilitate or inhibit regulation of vaccine  trials – Communities, politicians, etc.  2. How will you describe the role played by international stakeholders to facilitate or inhibit regulation of  vaccine trials - WHO, AVAREF, MSF, …  3. Have you ever felt the autonomy/independence of your organization or country has been under pressure  in relation to vaccine trials (proposed or in place) during public health emergencies?  4. What has been your organization’s approach to responding to pressures on its autonomy/independence?  Has this approach been effective in your view?  5. Does political pressure have a role to play in vaccine trial regulation during public health emergencies? |
| **Day 2** | |
| 9:40-10:10 | How have lessons from the time of the Ebola outbreak informed current procedures in vaccine trial regulation; requirement, process, payment, timelines, consent, and follow-up during trials |
|  | **Key questions to be addressed by speakers** |
|  | - Brief description of current guidelines for submission - How have current guidelines responded to lessons from the Ebola outbreak? Specific examples? - What more can be done to improve the processes |
| 10:30-10:45 | Presentation of vignette to guide group discussion: A new Ebola outbreak in West Africa |
|  | **VIGNETTE**: An outbreak of a new variant of Ebola has been reported in West Africa. This variant causes  severer disease and is highly fatal. Children and pregnant women are particularly at risk of severe disease and poor outcomes. The government has ordered all offices to be closed and workers to work from home. Pressure is mounting on the Government and the international community to mount an appropriate response within the shortest possible time.  Preliminary data suggest the currently available vaccines are ineffective in preventing disease from this variant. A candidate vaccine that is administered through intramuscular injection has been produced by a registered but less known company in Europe. Pre-clinical and phase 1 data are promising.  The company has approached regulators in Guinea and Sierra Leone to seek a way forward.  • Based on experiences during the Ebola outbreak, what are the key challenges you foresee with the review  and regulation of this trial.  • Identify **seven** key changes in your regulatory procedures (since the Ebola outbreaks) that has positioned  your institution to be able to respond to this challenge?  • Are these 7 changes (above) reflected in your current guidelines?  • What other changes would you recommend as necessary to better position your institution to respond to  this situation? |
| 15:30-16:00 | Brief reflections – How can regulatory agencies collaborate during public health emergencies – Intra-country and inter-country? |
|  | **Key questions to be addressed by speakers** |
|  | • Speakers’ discretion and ideas  • Suggestions to consider: Simultaneous submission? Online platform? Within the context of Mano River  Union? Or Ecowas? Annual meetings for experience-sharing? |
