# Supplementary material for: Regulator experiences of trials during Ebola epidemics in Sierra Leone, Guinea, and the Democratic Republic of the Congo
Source: Trop Med Int Health. 2025 Apr 3;30(6):539–46. doi: 10.1111/tmi.14111 (PMC12136929; doi:10.1111/tmi.14111)
Supplement: Supplementary file 3 — TABLE S3: Interview guides DRC. [file TMI-30-539-s003.docx]

**Supplementary Table 3: Interview guides Democratic Republic of Congo (DRC)_Guide to an In-depth interview with the people responsible for the regulation and deployment of vaccine trials in the DRC**

| This interview guide is structured as follows:  1. Introductory remarks  2. Maintenance  3. Concluding remarks | |
| --- | --- |
| 1. **Introduction** | |
|  | We'll start by introducing ourselves.  Hello, my name is ....., I'm a researcher at .... and I'm working on a European project called EBOVAC3 at the University of Antwerp and the University of Kinshasa.  The aim of this project is to conduct the latest clinical trials in Guinea, Sierra Leone, and the DRC for the licensing of an Ebola vaccine produced by Johnson & Johnson. As part of this project, there is a socio-cultural component which is interested, among other things, in describing the experience of ethical guidelines and the regulation of vaccine trials in the DRC during the latest outbreaks of EVM that have occurred in Equateur and Tshuapa Province since 2014. Specifically, the aim is to take stock of the current laws and regulations and standards relating to the importation, evaluation, and deployment of vaccines at national and provincial level. In addition to documenting the laws and regulations as well as the reports of ad hoc committees, workshops and meetings of the ethics committee, no objection notices, official documents and temporary authorizations for use that have been issued since the 2014 epidemic in Boende (7^ème^ epidemic reported in the DRC), we are seeking to determine what has been easy to do, what has been difficult to do and to analyse the reasons for resistance to changes in standards and regulations in relation to clinical trials during an epidemic |
| 1. **Maintenance** | |
| **Themes of the in-depth interview** | |
| ***Theme 1: General experience*** | - Could you tell me what position and role you currently hold on to the ethics committee (give the name of the ethics committee) ......? - What other affiliations/activities do you have outside the Ethics Committee? - How long have you been involved as a member of Ethics Committee X in assessing the protocols for setting up and rolling out vaccine trials in the DRC? - What diseases are you receiving vaccine trial protocols for? - For which phases of trials do you usually assess protocols in the ethics committee? - Compared with other health research protocols, have you received more or fewer protocols for vaccine trials since 2014 (West African epidemic)? - Under what circumstances (before, during or after an epidemic) did you receive protocols for vaccine trials? For what level of urgency? - What are the reasons for refusal? - Could you describe in simple terms the routine regulatory process for ethics committee approval of vaccine trial protocols in general? And more specifically in relation to Ebola? |
| ***Theme 2: Perception of ethical aspects during the epidemic(s)*** | - Were you involved in evaluating the protocol for setting up and deploying vaccine trials during the outbreak(s) of the last two MVE epidemics that took place in Equateur Province, respectively the 9^ème^ (2018) and 11^ème^ (2020) epidemics? - If so, can you please tell us about the experiences you had when you had to review and supervise vaccine trial protocols at that time?   (Emphasis on reports of meetings of the ethics committee, reports of monitoring missions)   - Compared with the situation before the Ebola epidemic in West Africa, have you noticed any major differences in the procedure for submitting vaccine trial protocols?   (Insist on the differences: urgency of the request? Time taken to examine the protocols. Over what period of time?)   - How did you deal with this situation? How did you prioritize other work? - Approximately how many protocols for Ebola vaccines have been submitted to you since 2014?   - How were they submitted to you?   (Emphasise the circumstances of this submission and the importance of the level of urgency)   - - How many were turned down? And why were they rejected?   (Emphasise the most significant change observed and the reasons for this change***)***   - At what stage of the vaccine trial is informed consent mandatory? Is informed consent still required during an epidemic when the vaccine is licensed? - Were there any ways of doing things during one of the epidemics that satisfied you or did not meet your ethical requirements? |
| ***Theme 3: WHO protocol and guidelines*** | In 2017, the WHO, via the Strategic Advisory Group of Experts on Immunisation (SAGE), recommended the distribution of a non-approved vaccine under the expanded access / compassionate use protocol.   - At the time, how did you feel about this? - How did the members of the Ethics Committee view this recommendation? - How has this recommendation affected your work? - How was this recommendation implemented during the 2018 epidemic in Mbandaka? - How can this recommendation be ethically combined with the implementation of a clinical trial? |
| ***Theme 4: Ethical aspects of the roll-out of vaccine trials*** | - What did you think of the ethical aspects of exporting biological samples?   (Emphasise the need for such exports of biological samples)   - - What was your role in monitoring this export of biological samples and what problems, if any, did you encounter?   - What do you think of the documentation processes for these sample shipments?   - Were there any ways in which researchers exported biological samples during epidemic outbreaks that met or failed to meet your ethical requirements? And how? - How have the protocols submitted to you dealt with the ethical aspects of excluding/including pregnant women and children in trials during the epidemic(s)? - Have you received an Ebola vaccine trial protocol that uses the age de-escalation approach to include children of different ages?   - If so, were the ethical aspects respected in this protocol?   - What did you think? |
| ***Theme 5: Revision of* guidelines** | - Following the experience of the Ebola epidemic in West Africa, have your national/provincial guidelines for conducting vaccine trials been revised? - If so, by whom? And by whom? - What role did your ethics committee play in revising the guidelines? - Are you satisfied with the revised guidelines? If so, why? If not, why not and what more needs to be done to improve the revision? - Was there any pressure on the ethics committees from the political/health/academic authorities to agree to set up a clinical trial? |
| ***Theme 6: Collaboration between vaccine protocol review bodies*** | It is suggested that vaccine protocol review bodies work together in the event of an emergency.   - What do you think of this suggestion? - What other examination bodies do you work with/need to work with? - What are your views on the current proposal for dialogue between the review bodies? - How can this dialogue be better implemented?   - For example, what kind of information can be shared? - What are the lessons learned/exchanges between countries (e.g. Guinea, Sierra Leone) in relation to ethical regulations in the field of Ebola (in general and during an epidemic)? - What are the advantages (benefits)/disadvantages of allowing a clinical trial to be set up during an epidemic? |
| 1. **Conclusion** | |
|  | After the in-depth interview session, the interviewer will ask the respondent if he/ she has anything to add and if there is anyone else, he/ she would be interested in meeting.  Before leaving the respondent, the interviewer will remind him/her that, in accordance with the principle of confidentiality, no one else will know that he/she has taken part in this research, apart from project staff.  Finally, the interviewer will thank the respondent for their time and valuable information before saying goodbye. |
