# Supplementary material for: Regulator experiences of trials during Ebola epidemics in Sierra Leone, Guinea, and the Democratic Republic of the Congo
Source: Trop Med Int Health. 2025 Apr 3;30(6):539–46. doi: 10.1111/tmi.14111 (PMC12136929; doi:10.1111/tmi.14111)
Supplement: Supplementary file 4 — TABLE S4: List of individual interviews DRC. [file TMI-30-539-s002.docx]

**Supplementary table 4 – List of individual interviews, May-June 2021, DRC**

| **N°** | **Site** | **Category** | **Name of the institution** | **Role** |
| --- | --- | --- | --- | --- |
| 1 | Kinshasa | Ethics committee member | Comité ethique ESP - Université de Kinshasa | Vice-president |
| 2 | Kinshasa | Health authority | Direction de la pharmacie et des médicaments (Ministère de la Santé Publique, Hygiène et Prévention) | Chief of Division Gestion des Médicaments |
| 3 | Kinshasa | Ethics committee member | CNES - Comité éthique national | President |
| 4 | Kinshasa | Ethics committee member | Comité éthique UPC (Université Protestante du Congo) | Secretary |
| 5 | Kinshasa | Health authority | Village Reach | Program Manager/Principal Investigator |
| 6 | Kinshasa | Health authority | Projet THIS | Project Manager |
| 7 | Kinshasa | Ethics committee member | Plateforme Comité Ethique de la Clinique Ngaliema | Doctor |
| 8 | Kinshasa | Health authority | Institut National de Recherche Biomédicale (INRB) | Head of the Virology Department |
| 9 | Kinshasa | Ethics committee member | Comité éthique ESP - Université de Kinshasa | Technical secretary |
| 10 | Kinshasa | Health authority | Département de pharmacovigilance, Université de Kinshasa | Professor in Pharmacovigilance |
| 11 | Boende | Health authority | Zone de Santé de Boende | Médecin Chef de Zone |
| 12 | Boende | Health authority | Centre de santé Lokolia | Infirmier Titulaire |
| 13 | Boende | Health authority | Organisation Mondiale de la Santé, Boende | Epidemiologist in charge of disease surveillance |
| 14 | Boende | Health authority | PDSS - Projet du Programme de Développement de Système de Santé de Boende | Provincial Director |
| 15 | Boende | Health authority | Projet Monkeypox | Project Coordinator |
| 16 | Boende | Health authority | Zone de Santé Boende | Coordinator of the Zone de Santé |
| 17 | Boende | Health authority | Centrale de Distribution des Médicaments de Boende | Director |
| 18 | Boende | Health professional | Zone de Santé de Boende | Technician (Laborantin) |
| 19 | Boende | Health authority | Zone de Santé de Boende | Doctor, Centre de Santé Lokolia |
| 20 | Boende | Health professional | Programme d'Evaluation des Vaccinations de Boende | Technician (Laborantin) |
| 21 | Mbandaka | Health authority | PEV - Programme Elargi de la Vaccination, Mbandaka | Doctor Head of the Antenne |
| 22 | Mbandaka | Health authority | Division Provinciale de la Santé | Epidemiologist |
| 23 | Mbandaka | Health authority | Division Provinciale de la Santé / Médecins Sans Frontières | Epidemiologist |
| 24 | Mbandaka | Health authority | PDSS - Projet du Programme de Développement de Système de Santé | Medical Analyst (Verificateur) |
| 25 | Mbandaka | Health professional | Laboratoire de Santé Publique, Mbandaka | Technician (Laborantin) |
| 26 | Mbandaka | Political authority | Gouvernement provincial de la Province de l'Équateur | Provincial Minister of Interior |
| 27 | Mbandaka | Political authority | Assemblée provinciale de la Province de l'équateur | Provincial deputy |
| 28 | Mbandaka | Health authority | Inspection Provinciale de la Santé de Mbandaka | Inspector |
| 29 | Mbandaka | Health professional | Laboratoire de Santé Publique, Mbandaka | Technician (Laborantin) |
| 30 | Mbandaka | Health authority | Zone de Santé de Mbandaka | Médecin Chef de Zone de Santé |
| 31 | Mbandaka | Health authority | Centre de Santé de Référence à Mbandaka | Médecin Directeur |
| 32 | Mbandaka | Health authority | Centre de Santé de Référence à Mbandaka | Infirmier Titulaire |
